# Conservative management of gastric ulcer penetration using a polyglycolic acid sheet: a case report

**DOI:** 10.1055/a-2791-5087

**Published:** 2026-02-17

**Authors:** Toshiyuki Wakatsuki, Tatsuhiro Go, Jun Harada, Hiroki Numoto, Yuta Nanimoto, Tomoko Sato

**Affiliations:** 1 38306 Department of Gastroenterology, Okayama Kyoritsu Hospital, Okayama, Japan

**Fig. 1:**
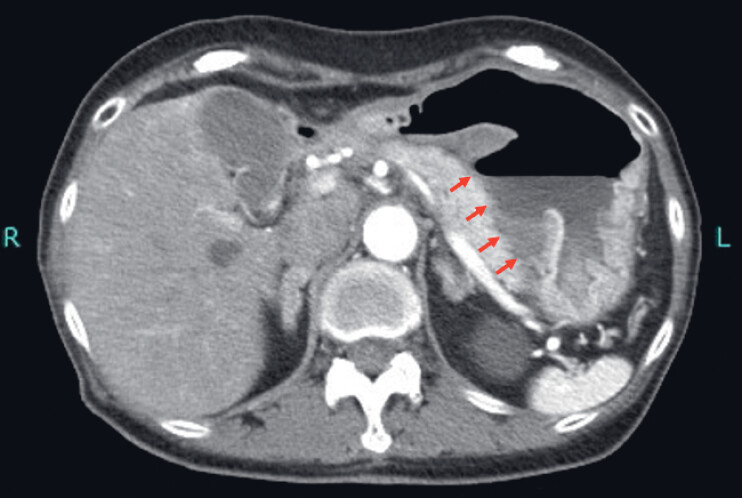
Computed tomography revealed a gastric ulcer penetrating into the pancreas (red arrow) with no free air within the abdominal cavity.

**Fig. 2:**
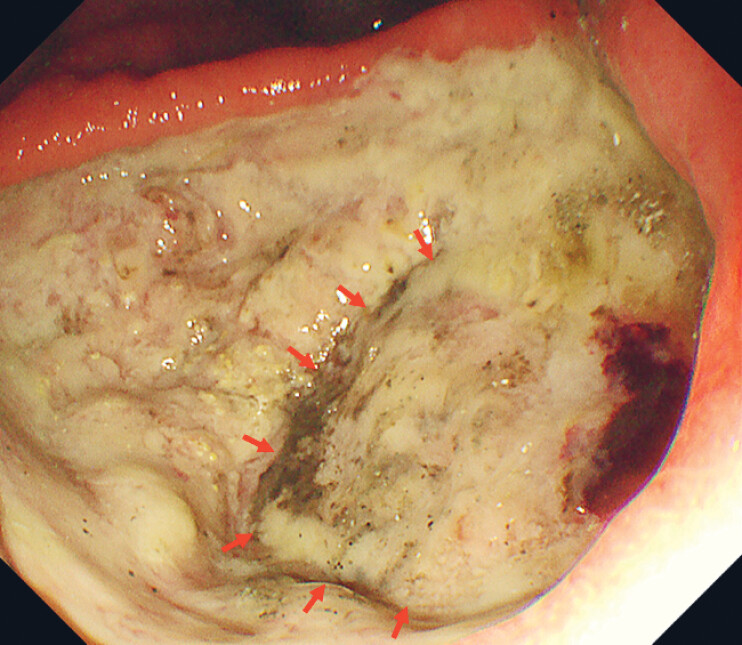
Esophagogastroduodenoscopy revealed an active gastric ulcer in the lesser curvature of the gastric angle. A region deeper than the base of other ulcers was observed, raising suspicion of penetration into pancreatic tissues (red arrow).

**Fig. 3:**
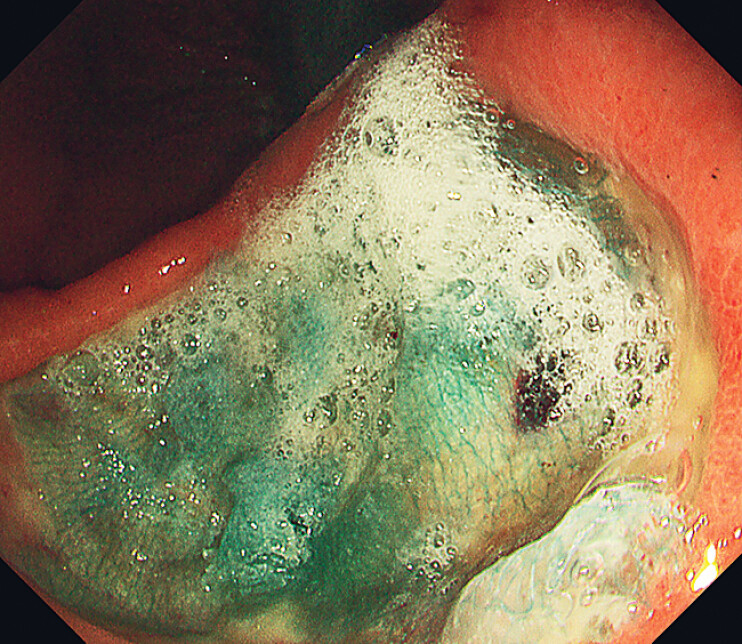
PGA sheets were used to cover the ulcers and were fixed in place with fibrin glue. PGA, polyglycolic acid.

**Fig. 4:**
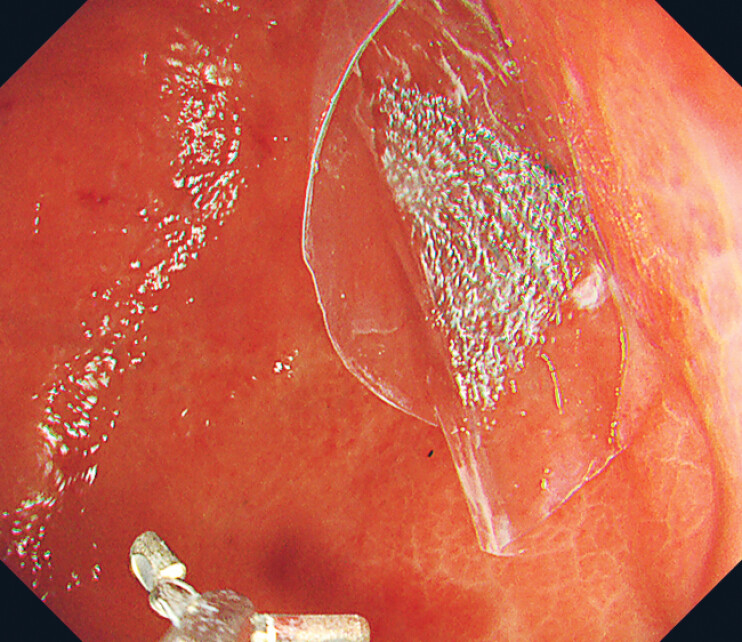
PGA sheets were delivered into the stomach using the Original OPP bags. OPP, oriented polypropylene; PGA, polyglycolic acid.

**Fig. 5:**
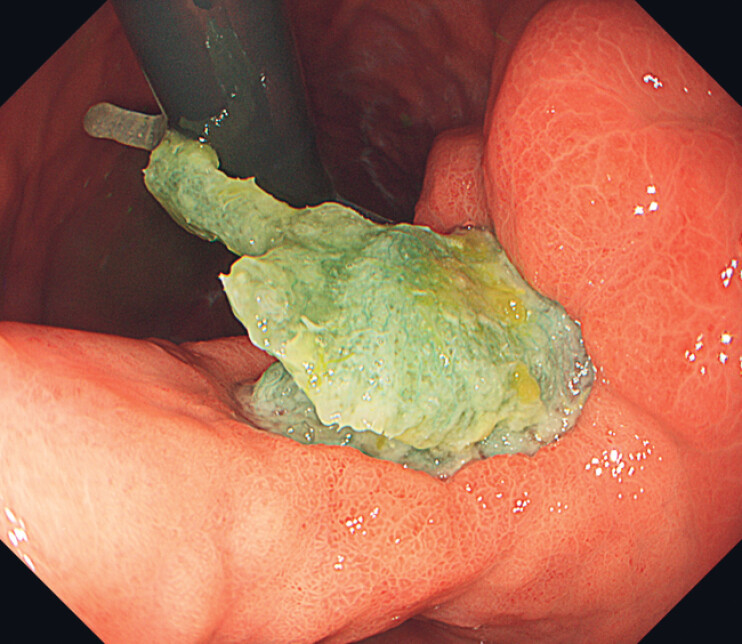
Ten days after the removal of the OPP bags, endoscopic examination revealed a reduction in ulceration, epithelial regeneration, and residual PGA sheets. OPP, oriented polypropylene; PGA, polyglycolic acid.

A 76-year-old woman was admitted with loss of appetite. Physical examination revealed no abnormalities, but blood tests showed mild anemia. Computed tomography revealed a gastric ulcer penetrating into the pancreas ( Fig. 1 ), without free air in the abdominal cavity. Esophagogastroduodenoscopy revealed an active gastric ulcer on the lesser curvature of the gastric angle and pancreatic tissue visible at the ulcer base, confirming penetration ( Fig. 2 ). She was diagnosed with gastric ulcer penetrating the pancreas and managed conservatively. A polyglycolic acid (PGA) sheet and fibrin glue were applied; custom-made oriented polypropylene (OPP) bags were used to deliver a PGA sheet into the stomach and cover the ulcer base ( Fig. 3 and Fig. 4 ; Video 1 ). We prepared original OPP bags for delivery by cutting commercialized ballpoint pen packs into 40-mm pieces. PGA sheets shrink in size and lose adhesive strength when exposed to saliva or mucus. Using OPP bags allows large PGA sheets to be stored in envelopes during transport, enabling rapid delivery and fixation in the stomach without exposing them to saliva or mucus. The bags were removed after the procedure. Ten days later, endoscopic examination revealed a reduction in ulceration, epithelial regeneration, and residual PGA sheets ( Fig. 5 ). Oral intake was resumed, and the patient was discharged 2 weeks later.

Endoscopic polyglycolic acid sheet application for gastric ulcer perforation.Video 1

Peptic ulcers can penetrate the bowel wall without causing free perforation or leakage into the peritoneal cavity, leading to atypical symptoms due to the involvement of adjacent organs. Although surgical intervention is usually required, conservative management was feasible in our case because there were no inflammatory findings or free air in the abdominal cavity. To our knowledge, this is the first reported case of a gastric ulcer penetrating the pancreas successfully treated with a PGA sheet to promote wound healing, resulting in favorable recovery. Previous reports have demonstrated the effectiveness of PGA sheets in treating gastrointestinal fistulas 1 2 3 , serving as a scaffold for tissue regeneration and inducing the growth of granulation tissue that covers perforations 4 . In cases of peptic ulcer penetration without free air, the endoscopic application of PGA sheets may be an alternative to surgery.

Endoscopy_UCTN_Code_TTT_1AO_2AN Endoscopy_UCTN_Code_TTT_1AO_2AO Endoscopy_UCTN_Code_CCL_1AB_2AC_3AZ
